# Evaluation of different recruitment and randomisation methods in a trial of general practitioner-led interventions to increase physical activity: a randomised controlled feasibility study with factorial design

**DOI:** 10.1186/1745-6215-15-134

**Published:** 2014-04-21

**Authors:** Fiona C Warren, Kate Stych, Margaret Thorogood, Deborah J Sharp, Marie Murphy, Katrina M Turner, Tim A Holt, Aidan Searle, Susan Bryant, Caroline Huxley, Rod S Taylor, John L Campbell, Melvyn Hillsdon

**Affiliations:** 1 Primary Care Research Group, University of Exeter Medical School, St Luke’s Campus, Heavitree Road, Exeter EX1 2LU, UK; 2 Department of Sport and Health Sciences, University of Exeter, St Luke’s Campus, Heavitree Road, Exeter EX1 2LU, UK; 3 Warwick Medical School, University of Warwick, Gibbet Hill Campus, Coventry CV4 7AL, UK; 4 Centre for Academic Primary Care, School of Social and Community Medicine, Canynge Hall, 39 Whatley Road, University of Bristol, Bristol BS8 2PS, UK; 5 Sport & Exercise Sciences Research Institute, Jordanstown Campus, Shore Road, Newtownabbey, University of Ulster, BT37 0QB, UK; 6 Department of Primary Care Health Sciences, Radcliff Observatory Quarter, Woodstock Road, Oxford University, Oxford OX2 6GG, UK

**Keywords:** Primary care research, Physical activity, Complex interventions, Clinical trials

## Abstract

**Background:**

Interventions promoting physical activity by General Practitioners (GPs) lack a strong evidence base. Recruiting participants to trials in primary care is challenging. We investigated the feasibility of (i) delivering three interventions to promote physical activity in inactive participants and (ii) different methods of participant recruitment and randomised allocation.

**Methods:**

We recruited general practices from Devon, Bristol and Coventry. We used a 2-by-2 factorial design for participant recruitment and randomisation. Recruitment strategies were either opportunistic (approaching patients attending their GP surgery) or systematic (selecting patients from practice lists and approaching them by letter). Randomisation strategies were either individual or by practice cluster. Feasibility outcomes included time taken to recruit the target number of participants within each practice. Participants were randomly allocated to one of three interventions: (i) written advice (control); (ii) brief GP advice (written advice plus GP advice on physical activity), and (iii) brief GP advice plus a pedometer to self-monitor physical activity during the trial. Participants allocated to written advice or brief advice each received a sealed pedometer to record their physical activity, and were instructed not to unseal the pedometer before the scheduled day of data collection. Participant level outcomes were reported descriptively and included the mean number of pedometer steps over a 7-day period, and European Quality of Life (EuroQoL)-5 dimensions (EQ-5D) scores, recorded at 12 weeks’ follow-up.

**Results:**

We recruited 24 practices (12 using each recruitment method; 18 randomising by cluster, 6 randomising by individual participant), encompassing 131 participants. Opportunistic recruitment was associated with less time to target recruitment compared with systematic (mean difference (days) -54.9, 95% confidence interval (CI) -103.6; -6.2) but with greater loss to follow up (28.8% versus. 6.9%; mean difference 21.9% (95% CI 9.6%; 34.1%)). There were differences in the socio-demographic characteristics of participants according to recruitment method. There was no clear pattern of change in participant level outcomes from baseline to 12 weeks across the three arms.

**Conclusions:**

Delivering and trialling GP-led interventions to promote physical activity is feasible, but trial design influences time to participant recruitment, participant withdrawal, and possibly, the socio-demographic characteristics of participants.

**Trial registration number:**

ISRCTN73725618.

## Background

A physically active lifestyle helps in the prevention and management of over 20 chronic conditions including coronary heart disease, stroke, diabetes, some cancers, osteoporosis and depression, and yet the prevalence of physical activity is low [1]. In the United Kingdom (UK), the National Institute for Health and Care Excellence (NICE; formerly the National Institute for Health and Clinical Excellence) has advised that all General Practitioners (GPs) should identify inactive patients and advise them to increase their physical activity [2]. Furthermore, the government’s physical activity strategy committed GPs to widening screening for physical inactivity using the General Practice Physical Activity Questionnaire (GPPAQ) and led to the development of the Primary Care Physical Activity Care Pathway, ‘Let’s Get Moving’ [3,4]. Despite advice and guidelines for GPs to promote physical activity, there is a lack of evidence to inform the optimal nature of an effective primary care-based intervention and, specifically, a GP-led intervention [5-7]. Although a randomised controlled trial (RCT) of the clinical effectiveness of a brief GP-led intervention could provide an answer as to whether such interventions can increase patient physical activity levels, there are a number of methodological and practical uncertainties that preclude immediate progress to a full RCT.

The success of research in primary care depends on the willing participation and cooperation of patients, GPs, and other primary care staff. It is well documented that many RCTs fail to recruit to target, potentially resulting in loss of statistical power, a requirement for additional resources, and delays in the dissemination of results [7,8]. A review of RCTs funded by two UK funding agencies found that only 31% of trials recruited to target [9]. A more recent review reported that 29% of UK primary care trials recruited as planned, with 35% requiring up to 50% more time than planned and 35% requiring even longer [10]. Delays to patient recruitment in primary care trials can operate both at the level of the practice and the patient. Recruitment of practices that are representative of practices across a region or the country is essential for the external validity of research, but practice recruitment presents many difficulties, in terms of the time taken to recruit sufficient practices, and whether sufficient practices can be recruited at all. Compared with studies of patient recruitment to trials, far fewer studies have examined causes of delays to practice recruitment [10], either at the organisational level (for example, approvals from Trust research and development departments, National Health Service (NHS) ethics committees and academic departments), or at the practice level. Within practices, existing research has focused on clinicians’ willingness to participate in trials and then their subsequent recruitment of participants [11,12]. No strong, consistent, associations have been found between clinician factors and their willingness to participate in trials, although potential areas for improvement include reinforcement of clinician and patient benefit and better communication of the research method [12].

The Doctor-DELivered PHysical activity Intervention (DDELPHI) study was a three-arm feasibility RCT to inform the design of a full RCT to test the clinical effectiveness of two GP-led interventions promoting physical activity in inactive participants. We used a factorial design to evaluate the effect on recruitment and retention of two trial design factors: (i) random allocation (by practice (cluster) or by individual participant); and (ii) recruitment of participants (opportunistically from the waiting room or systematically by written invitation).

A cluster randomised design simplifies the delivery of the intervention and recruitment of participants. Practices were preferred as the cluster, rather than individual GPs, to avoid the potential difficulty of having very small cluster sizes. Also, cluster randomisation protects against the possibility of contamination, which is an issue with individual randomisation. Contamination may occur at the level of the GP, for example, if GPs erringly provide verbal advice to participants allocated to receive only written advice, or at the level of the participant, for example if participants within the same practice confer about their (different) interventions (this could occur using individual randomisation or clustering by individual GP). However, this consideration needs to be balanced with the fact that cluster RCTs generally require larger sample sizes than trials randomised by individual participant [13]. In this feasibility study, it was possible to administer the intervention both at the level of the individual participants and at the level of the practice, allowing comparison of both delivery types. We have also assessed opportunistic and systematic methods of participant recruitment.

In this paper, we compare the two design factors in terms of trial feasibility: rates of practice and participant recruitment, degree of baseline balance in socio-demographic factors, and loss to follow-up. These findings are central to the design and sample size calculation of a future definitive trial. The trial results are also reported descriptively, to demonstrate feasibility of collecting the required data, and to facilitate presentation of intra-cluster correlation coefficients (ICCs), which may be used in the design of future studies.

## Methods

### Study design

**Figure 1:**
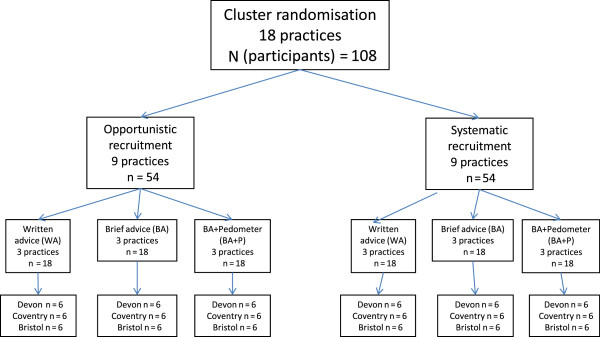
Practice recruitment schedule by participant randomisation and recruitment factors.

The DDELPHI feasibility study was conducted between June 2010 and September 2011. Ethical approval for the study was obtained from the South West 1 Research Ethics Committee in April 2010 (reference number: 10/H0203/18). In addition to random allocation to one of the three treatment arms, we used a 2 × 2 factorial design to distribute practices and participants across two trial design factors: cluster versus individual allocation and systematic versus opportunistic recruitment (see Figure 1).

We randomly assigned 24 practices (8 practices in each of 3 geographical regions (Bristol, Devon and Coventry)) in a 3:1 ratio to cluster (practice) allocation or individual allocation, and in a 1:1 ratio to opportunistic or systematic recruitment. The differential allocation ratio with regard to randomisation method was due to the need to ensure even numbers of practices and participants in each of the three arms across the cluster randomised practices. Based on a planned recruitment of a total of 144 participants (6 from each of 24 practices) we sought to allocate an equal number of participants to each of the three treatment arms (that is, 48 per arm). We randomised by practice at 18 of the 24 practices; of these 18 practices, 9 recruited participants opportunistically, while 9 recruited participants systematically, with the aim of recruiting 54 participants by each recruitment method within cluster randomisation. We randomised participants individually at 6 practices, with opportunistic recruitment and systematic recruitment at 3 practices for each method; this procedure was designed to result in recruitment of 18 participants by each recruitment method within individual randomisation. In each region, a single researcher was responsible for practice and participant recruitment.

### Practice eligibility and recruitment factors

In each region, the primary care research network (PCRN; funded by the National Institute for Health Research; NIHR) provided a list of practices that could be approached with regard to participating in the study. No formal eligibility criteria (for example, regarding list size or deprivation) were specified, although these practice characteristics were described in order to explore their potential influences on practice recruitment, and to assess comparability of these characteristics by design factors.

### Participant inclusion factors

Participants were eligible if they were aged 40 to 74 years (the age range recommended by UK Department of Health guidelines for vascular screening [14]), were classified as inactive by the GPPAQ [4], and were able to walk continuously for 5 minutes without undue fatigue or discomfort. The GPPAQ classifies a person as ‘inactive’ if he/she has a sedentary job and does not do any physical exercise or cycling; furthermore, 74 years is the upper age limit recommended for use of the GPPAQ.

### Trial design factors

Two methods of random allocation to treatment arm were tested: (i) cluster allocation - randomly allocating participants at the practice level to one of the two intervention arms or control - and (ii) individual allocation - individually randomising participants within practices to one of the two intervention arms or control.

Two recruitment strategies were tested: (i) ‘opportunistic’ recruitment and (ii) ‘systematic’ recruitment. ‘Opportunistic’ recruitment required a researcher to approach patients in the practice waiting room who were attending routine appointments with a GP. A simple two-question screening established whether the patient was aged 40 to 74 years and could walk continuously for 5 minutes without undue fatigue or discomfort. If the patient met these criteria, the potential participant was given a participant information sheet and requested to complete the GPPAQ. If eligible (that is, classed as inactive according to the GPPAQ), the participant provided written informed consent to the researcher before being called to see the GP. If the participant was being randomised individually, the eligibility was established prior to the participant’s selection of a sealed envelope containing the intervention allocation.

The second method (‘systematic’) required GPs to select potentially eligible patients (aged 40 to 74) from a list of patients (provided by practice staff) registered with the practice. No further eligibility criteria were provided to GPs in addition to the age range, as further eligibility would be determined if the patient expressed interest in participation. A letter of invitation, participant information sheet, GPPAQ and pre-paid envelope were then sent from the practice to selected patients. Interested patients were asked to complete an expression of interest form and GPPAQ, and return these to the research team. If patients were eligible (as above), research staff telephoned them to answer any questions and request they make an appointment with their GP. GPs were sent details of all patients willing to participate, and obtained their written informed consent on attendance at the appointment. If the participant was individually randomised, he/she randomly selected an envelope containing arm allocation at the GP appointment. Ineligible patients were sent a letter informing them of their ineligibility and thanking them for their interest in the study.

### Randomisation procedure and concealment

Following agreement to participate, practices were allocated to one of four options (that is, one of the three interventions or individual participant randomisation) in a 1:1:1:1 ratio using a computer generated random list, with stratification by location and recruitment method. Participants who were recruited opportunistically and randomised individually selected a sealed envelope (containing treatment arm allocation) to give to the GP at the start of their appointment. The envelope contained both the participant’s arm allocation and instructions for the GP on what intervention to deliver. This procedure ensured that both the researcher and participant were unaware of the intervention the participant would receive prior to the participant’s agreement to enter the trial. For each practice conducting opportunistic recruitment and individual randomisation, six envelopes were available, with each intervention contained within two envelopes. For participants who were recruited systematically and randomised individually, randomisation was by computer generated list, in a 1:1:1 ratio across the three interventions within each practice. The participant’s GP received a pack including the participant’s intervention allocation prior to the scheduled consultation. All computer generated randomised sequences were performed by a statistician (RST) who was not involved with the analyses.

### Treatment arms

Three treatment arms were investigated; all interventions were delivered during a GP face-to-face consultation within the practice. In the control arm (Arm A; written advice), participants were given (during the consultation at which the intervention was delivered): (i) written information on physical activity and its benefits, plus information on local opportunities for participation in physical activity (but no specific guidance on the duration or type of activity that would be beneficial); (ii) a pedometer that stores the most recent 7 days of steps (New Lifestyles NL-800), which was sealed so that participants could not view the readings during the intervention, and written information on how to use it; and (iii) an envelope containing a baseline questionnaire, pre-paid return envelope (for the questionnaire and pedometer), a summary of the study, and a participant information sheet. In the first of the two intervention arms (Arm B: brief GP advice), participants received the same items and information as for Arm A and were also advised by their GP to “walk at least a mile per day (15 to 20 minutes) at a brisk to fast pace, each day of the week”. In the second intervention arm (Arm C: brief GP advice plus pedometer), participants received the same items, written physical activity information and verbal advice as in Arm B. However, for Arm C, the pedometer was unsealed (allowing participants to view the display of the number of steps taken), and participants were given written information about how to use the information to self-monitor their physical activity levels. They were specifically guided to work towards 10,000 steps per day (approximately equivalent to 150 minutes per week; [15]). Based on the recorded steps, the written information provided details on how to set intermediate goals to assist gradual progression towards the 10,000 steps per day target. All interventions were delivered within the context of a GP consultation; no formal measurement of the amount of time GPs required to deliver the intervention was made.

All participants were asked to wear the pedometer for 7 days, commencing the day after their appointment with the GP. On the 8^th^ day, researchers telephoned the participants to collect pedometer data (to prevent pedometer data from being automatically erased), and participants in Arms A and B were asked to return their pedometer and questionnaire in the pre-paid envelope. Participants in Arm C kept their pedometers over the 12-week period. Participants in Arms A and B were instructed not to remove the seal prior to the end of the intervention period. Although we cannot be sure that participants did not unseal the pedometer before the scheduled time, we believe that in general participants did not do this, as many of them had difficulty removing the seal and opening the pedometer when the researchers contacted them by telephone to collect their data.

All participants were sent a repeat of the baseline questionnaire at 12 weeks’ follow-up, along with a sealed pedometer for those in Arms A and B, and a pre-paid envelope for return. Participants were also requested to wear their pedometers for 7 consecutive days from a specified date; a researcher then telephoned the participant on Day 8 to collect the pedometer data and ask participants to return the questionnaire. Participants were thanked for taking part in the study and invited to keep the pedometer as a ‘thank-you’ for their participation.

### Feasibility outcomes

Feasibility outcomes included: (i) time to practice recruitment (number of days from date of initial invitation to expression of interest (EOI)) by practice characteristics; list size: small (<3,500 registered patients), medium (3,500 to 8,000), large (>8,000); and deprivation (deprivation score using the Index of Multiple Deprivation (IMD) 2007, derived from The Network of Public Health Observatories [16]); (ii) time to participant recruitment (number of days from date of recruitment of first participant to recruitment of final participant); and (iii) level of loss to follow-up (defining loss to follow-up as failure to provide complete data on trial outcomes at 12 weeks follow-up). In addition, we collected participant socio-demographic data at baseline through the participant questionnaire or from practice records.

### Participant level outcomes

The primary participant level outcome was predefined in the protocol for the study and was the average (mean) number of steps walked per day by the participant, as recorded by the pedometer. This outcome was selected as the primary outcome at the participant level, as it was objectively measured, and would be the intended primary outcome for a fully powered trial. The primary outcome was measured twice - once over a 7-day period at the beginning of the study and again in the 7-day period at 12 weeks post-randomisation. Only participants who had a recorded step count of at least 1,000 on at least 4 days out of 7 [17] were included in calculating the primary outcome (for participants who did not meet this criterion, the primary outcome was recorded as ‘missing’). A recorded step count of less than 1,000 steps in one day may be indicative of inaccurate data (for example, pedometer malfunction or failure to wear the pedometer for the full day). Sedentary activities alone would be associated with a step count of at least 2,500 [15]; hence, only steps recorded on days where the total was at least 1,000 (eligible days) contributed to the total sum of steps. Thus, the primary outcome (mean number of steps per day) was defined as the total sum of steps walked (on eligible days) divided by the number of eligible days (for participants with at least 4 eligible days). Secondary outcomes included: physical activity commitment measured on a three-item self-rating scale with responses ranging from 0 to 10 [18], and health-related quality of life using the European Quality of Life (EuroQoL)-5 dimensions (EQ-5D; [19]). Both secondary outcomes were obtained from participant questionnaires at baseline and at 12 weeks. EQ-5D index scores were calculated using the Dolan algorithm for the UK population [20].

### Sample size and data analysis

The intended sample size of 144 participants would have allowed us to detect differences in recruitment and follow-up rates between design factors of 22% or larger (at 80% power and 5% alpha). To assess the impact of design factors, we report the mean difference and 95% confidence intervals (CIs) in feasibility outcomes by allocation method and recruitment process. Given the feasibility objective of this study, the participant level outcomes are reported descriptively at baseline and follow-up for each of the three treatment groups. The key objective of this feasibility study was not to inferentially compare participant level outcomes, so no formal sample size and power calculations were undertaken with regard to these outcomes. The ICC is reported for the primary trial outcome (mean number of steps recorded by pedometer) and for the EQ-5D, measured at 12-weeks’ follow-up.

## Results

### Practice characteristics by design factors

The practice characteristics were generally well balanced across both recruitment and randomisation design factors (Table 1). However, there was some imbalance in the practices by recruitment method with regard to practice deprivation. There were no practices allocated to opportunistic recruitment in the least deprived quartile, and also relatively fewer large practices by comparison to the group of practices performing systematic recruitment. Practices allocated to individual randomisation also had a relatively greater proportion of small practices, compared with the cluster randomised group.

**Table 1:** Characteristics of recruited practices by design factor

**Practice characteristics by participant recruitment method**		
	Opportunistic (N = 12)	Systematic (N = 12)
Practice list size^a^ (n (%))		
Small	4 (33)	3 (25)
Medium	5 (42)	3 (25)
Large	3 (25)	6 (50)
Practice deprivation score (n (%))		
Least deprived quartile	0 (0)	3 (25)
Central two quartiles	9 (75)	6 (50)
Most deprived quartile	3 (25)	3 (25)
**Practice characteristics by participant randomisation method**		
	Cluster (N = 18)	Individual (N = 6)
Practice list size^a^ (n (%))		
Small	4 (22)	3 (50)
Medium	7 (39)	1 (17)
Large	7 (39)	2 (33)
Practice deprivation score (n (%))		
Least deprived quartile	2 (11)	1 (17)
Central two quartiles	11 (61)	4 (67)
Most deprived quartile	5 (28)	1 (17)

^a^Small: <3,500; Medium: 3,500 to 8,000; Large >8,000.

### Participant characteristics by design factors

Participants recruited opportunistically included a larger proportion that had no household vehicle and a smaller proportion that had a degree or equivalent qualification (Table 2). This group also had a lower baseline mean for both steps per day and EQ-5D score. Overall, there was good balance in socio-demographic factors by randomisation method (data not shown).

**Table 2:** Participant demographic characteristics by recruitment method

	**Opportunistic recruitment (N = 73)**	**Systematic recruitment (N = 58)**
Gender (n (%))		
Male	49 (67.1)	39 (67.2)
Female	24 (32.9)	19 (32.8)
Age (mean (sd), n)	59.5 (9.8), 61	60.4 (8.7), 56
BMI (mean (sd), n)	27.1 (5.3), 59	27.0 (4.8), 55
Smoking status (n (%))		
No	51 (83.6)	50 (90.9)
Yes	10 (16.4)	5 (9.1)
Ethnicity (n (%))		
White	57 (95.0)	53 (94.6)
Other ethnicity	3 (5.0)	3 (5.4)
How many cars or vans are available for use by you and other people in your household? (n (%))		
0	11 (18.0)	6 (10.7)
1 or more	50 (82.0)	50 (89.3)
Highest educational qualification (n (%))		
University degree or equivalent	16 (26.7)	19 (33.9)
No qualifications or qualifications below degree	44 (73.3)	37 (66.1)
I am currently trying to increase the amount of physical activity that I do (scale of 0 to 10; mean (sd), n)	5.4 (2.7), 61	5.5 (2.8), 56
Baseline pedometer readings over 7 days^a^: mean number of steps (mean (sd), n)	6704 (2821), 56	8310 (2532), 53
EQ-5D index (mean (sd), n)	0.75 (0.23), 60	0.87 (0.14), 56

^a^Mean number of steps calculated as total number of steps recorded by pedometer divided by number of days (including only days with at least 1,000 steps recorded), for participants with at least 1,000 steps for 4 days or more out of 7 days. BMI, body mass index; EQ-5D, European Quality of Life-5 dimensions; sd, standard deviation.

### Time to practice recruitment by practice characteristics

Time to practice recruitment, from initial invitation to participate in the study to EOI, was not associated with the deprivation level of the practice. However, practices with a small list size (<3,500 patients) took on average a longer period of time to express interest compared with medium (3,500 to 8,000 patients) or large practices (>8,000 patients), although comparisons were not statistically significant (Table 3).

**Table 3:** Practice recruitment timeframe

	**All sites combined**	**Small versus Medium**	**Small versus Large**	**Medium versus Large**
Days from date of initial invitation to date of EOI Practice list size^a^ Median (IQR), n; mean (sd) :				
Small	56 (15, 59), 7; 40.7 (22.1)			
Medium	24.5 (14.25, 42), 8; 26.9 (17.2)			
Large	26 (20.5, 37.5), 9; 29.6 (13.6)			
Between-group mean difference (95% CI)		13.8 (-8.1; 35.8)	11.2 (-8.0; 30.4)	-2.7 (-18.6; 13.3)
		**least deprived quartile versus central two quartiles**	**least deprived quartile versus most deprived**	**central two quartiles versus most deprived quartile**
Days from date of initial invitation to date of EOI				
Median (IQR), n; mean (sd):				
Least deprived quartile	26 (22, 27), 3; 25.0 (2.6)			
Central two quartiles	24 (18, 57), 15; 33.6 (20.0)			
Most deprived quartile	26.5 (15, 49.5), 6; 31.2 (17.2)			
Between-group mean difference (95% CI)		-8.6 (-20.0; 2.8)^b^	-6.2 (-24.2; 11.9)^b^	2.4 (-17.1; 22.0)

^a^Small: <3,500; Medium: 3,500 to 8,000; Large >8,000.^b^95% CI derived from *t*-test with unequal variances. CI, confidence interval; EOI: Expression of interest; IQR, interquartile range; sd, standard deviation.

### Time to participant recruitment and loss to follow-up by design factors

Time from recruitment of the first participant within each individual practice to recruitment of the final participant is shown in Table 4 by randomisation and recruitment method. There was no difference in the mean time of participant recruitment by randomisation method (0.1 day, 95% CI -63.3; 63.5). However, opportunistic recruitment was associated with significantly faster participant recruitment than systematic recruitment (-54.9 days, 95% CI -103.6; -6.2).

**Table 4:** Within-practice participant recruitment timeframe by participant recruitment/randomisation factors

	**Randomisation method**	
	**Practice (cluster)**	**Individual**
Days from date of recruitment of first participant to recruitment of final participant by randomisation method (mean (sd), n)	58.1 (55.3), 18	58.0 (78.2), 5
Between-group mean difference (95% CI)	0.1 (-63.3; 63.5)	
	**Recruitment method**	
	**Opportunistic**	**Systematic**
Days from date of recruitment of first participant to recruitment of final participant by recruitment method (mean (sd), n)	31.8 (34.2), 12	86.7 (68.0), 11
Between-group mean difference (95% CI)^a^	-54.9 (-103.6; -6.2)	

^a^95% CI derived from t-test with unequal variances. CI, confidence interval; sd, standard deviation.

Participants recruited opportunistically were more likely to be lost to follow-up compared with those recruited systematically (Table 5). A greater proportion of participants allocated by cluster randomisation were lost to follow-up compared with those randomised individually, but the mean difference in proportions lost to follow-up between the two allocation methods was smaller than that between recruitment methods, and not statistically significant.

**Table 5:** Participant withdrawals and losses to follow-up by participant recruitment and randomisation method

**Intervention arm**			
	**Written advice (N = 44)**	**Brief advice only (N = 42)**	**Brief advice plus pedometer (N = 45)**
Withdrawn/lost to follow-up^a^ (n, %)	8 (18.2)	10 (23.8)	7 (15.6)
**Recruitment method**			
	**Opportunistic (N = 73)**	**Systematic (N = 58)**	
Withdrawn/lost to follow-up^a^ (n (%))	21 (28.8)	4 (6.9)	
% mean between group difference (95% CI)	21.9 (9.6; 34.1)		
**Randomisation method**			
	**Cluster (N = 102)**	**Individual (N = 29)**	
Withdrawn/lost to follow-up^a^ (n (%))	22 (21.6)	3 (10.3)	
% mean between group difference (95% CI)	11.2 (-2.4; 24.9)		

^a^Participant is considered withdrawn/lost to follow-up if the follow-up questionnaire and/or pedometer is not returned at 12-week follow-up. CI, confidence interval; sd, standard deviation.

### Participant disposition over the trial

**Figure 2:**
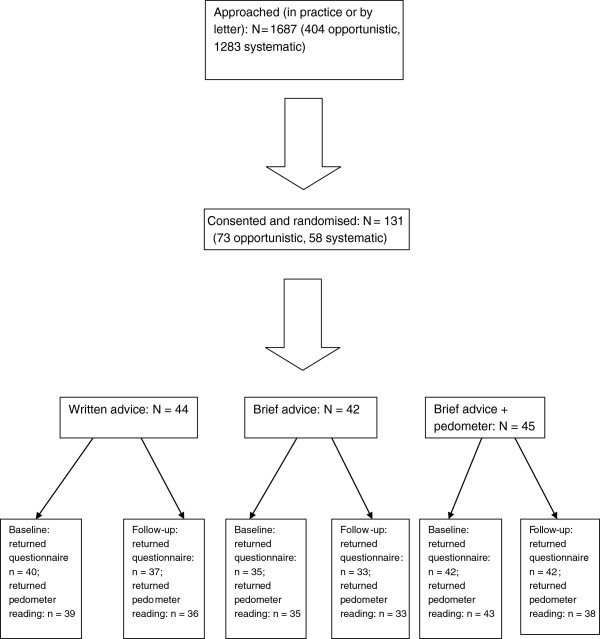
Participant flow diagram across all sites and recruitment types.

Participant flow through the trial, combining all three locations and all recruitment and randomisation factors, by trial arm, is shown in Figure 2. Of the 131 participants who provided consent to participate in the trial and were randomised, 73 were recruited using the opportunistic method (18.1% of those approached) and 58 using the systematic method (4.5% of those approached). At baseline, 114 participants (87.0%) returned both the questionnaire and pedometer, while at 12-week follow-up, 106 participants (80.9%) returned the questionnaire and pedometer. Loss to follow-up was similar across all intervention arms (Table 5).

### Comparison of participant level outcomes across treatment arms

There was a good balance in practice and participant characteristics across the three arms (data not shown). Whilst there appeared to be good balance at baseline across the three arms in terms of EQ-5D scores and commitment to change physical activity, the average level of pedometer-assessed activity in the two brief advice arms was higher than that seen in the control arm (Table 6). The ICC (by practice) for the average number of steps at follow-up was 0.106 (95% CI 0.000; 0.286). For EQ-5D, the ICC at follow-up was 0.114 (95% CI 0.000; 0.292). There was no clear pattern of change in outcomes from baseline to 12 weeks across the three arms. Whilst the level of commitment to change physical activity showed some increase following brief GP advice (Arm B), it was also increased in the written advice arm (Arm A), and lower in the brief GP advice plus pedometer arm (Arm C). Health-related quality of life assessed by EQ-5D remained unchanged across the three trial arms. Mean pedometer-assessed activity increased in the brief GP advice plus pedometer arm (Arm C), and written advice arm (Arm A), but showed a slight reduction in the brief GP advice arm (Arm B).

**Table 6:** Physical activity commitment, EQ-5D and pedometer readings at baseline and follow-up by treatment arm

	**Written advice (N = 44)**	**Brief advice only (N = 42)**	**Brief advice plus pedometer (N = 45)**
**Baseline**			
I am currently trying to increase the amount of physical activity that I do (scale of 0 to 10; mean (sd), n)	5.80 (2.66), 40	5.09 (2.90), 35	5.45 (2.66), 42
EQ-5D index (mean (sd), n)	0.79 (0.22), 40	0.85 (0.15), 34	0.80 (0.22), 42
Baseline pedometer readings over 7 days^a^: mean number of steps (mean (sd), n)	7074 (2531), 36	7860 (2675), 33	7545 (3110), 40
**Follow-up**			
I am currently trying to increase the amount of physical activity that I do (scale of 0 to 10; mean (sd), n)	6.08 (2.55), 37	5.70 (2.46), 33	5.29 (2.89), 42
EQ-5D index (mean (sd), n)	0.80 (0.24), 37	0.85 (0.20), 32	0.77 (0.27), 40
Follow-up pedometer readings over 7 days^a^: mean number of steps (mean (sd), n)	7,576 (3,101), 36	7,575 (2,918), 33	8,371 (3,069), 37

^a^Mean number of steps calculated as total number of steps recorded by pedometer divided by number of days (including only days with at least 1,000 steps recorded), for participants with at least 1,000 steps for 4 days or more out of 7 days. EQ-5D, European Quality of Life-5 dimensions; sd, standard deviation.

## Discussion

Our study shows that it is feasible to undertake an RCT of two interventions of brief advice on physical activity compared with written advice in a primary care setting. Whilst there appeared to be no impact of randomised allocation method (practice (cluster) level versus individual participant level) on time taken to recruit participants or loss to follow-up, an opportunistic recruitment method (approaching patients in the waiting room who were attending routine GP appointments) more than halved mean participant recruitment time compared with a systematic recruitment approach (GPs selecting eligible patients from practice lists). However, opportunistically recruited participants were on average four times more likely to withdraw or be lost to follow-up, therefore threatening the internal validity of the trial. Hence, the recruitment method has the potential to play a significant role in time taken to recruit participants, and may also influence the demographic characteristics of the sample. Such considerations may support the use of more than one recruitment method to facilitate the recruitment of a more diverse participant population.

Our finding that the opportunistic method of recruitment led to faster recruitment of the sample size, but with greater risk of drop-out or loss to follow-up, may be subject to confounding. Participants recruited opportunistically appeared to be in poorer general health compared with participants recruited systematically, and there was some evidence to indicate that opportunistic recruitment facilitated recruitment of participants from lower socio-economic backgrounds. Participants recruited whilst waiting to attend a GP appointment are likely, in general, to have a different current health status compared with those receiving an invitation by letter. Participants recruited opportunistically were less physically active than participants recruited systematically, and therefore had more to gain from increased physical activity, although this has to be balanced against the higher drop-out rate. Participants recruited systematically had more time to consider participating in a trial; giving increased consideration to deciding whether to participate may have led to the lower rate of drop-out. Perhaps surprisingly, commitment to change physical activity between the two recruitment methods was not different. It was expected that the postal method of recruitment, which allowed participants more time to reflect on the decision to participate, would have led to more motivated and confident participants than the opportunistic method. Hence, recruitment methods should take into account the desired demographic characteristics of the target population, balanced against the fact that more participants may withdraw when recruited opportunistically. In actuality, the advantage of faster participant recruitment using opportunistic methods may be somewhat offset with regard to the disadvantages of increased withdrawal and the subsequent necessity to recruit more participants to achieve the required sample size. Ideally, a balance would be found between recruitment across a wide range of the target population, to promote external validity, and achieving low attrition, thus protecting internal validity. A combination of opportunistic and systematic recruitment methods may be useful to obtain such a balance; awareness of the increased propensity of withdrawal of participants recruited opportunistically may lead to increased measures to prevent loss to follow-up for these participants.

As our study sought to test feasibility, it was not formally powered to detect a statistically significant difference between treatment arms in participant level outcomes. Nevertheless, we found no clear evidence of a consistent benefit of either of the two brief advice physical activity interventions (with or without pedometer feedback) over the control intervention in terms of participants’ commitment to increase physical activity, health-related quality of life (EQ-5D score) or mean pedometer counts at 12 weeks’ follow-up compared with baseline.

With regard to future sample size calculations, our estimations of the ICC for both mean number of steps and EQ-5D (both outcome measures that could be used in calculation of the sample size for a fully powered RCT) are potentially useful should a cluster randomised design be chosen, although the confidence intervals were wide, reflecting the small number of clusters on which to base the estimations.

### Comparison with previous studies

To the authors’ knowledge this is the first RCT of a physical activity promotion intervention that has formally compared the effects of different recruitment methods [11]. The descriptive data on the time to recruit different types of practice and the time taken to recruit participants via different methods are valuable in the planning of future RCTs. RCTs designed to examine the effects of interventions to promote physical activity during routine GP appointments need to test interventions in settings as close as possible to ‘normal’ practice conditions to ensure external validity. This study has shown that not only is it possible to recruit participants opportunistically while waiting for routine appointments, but also it is quicker than recruitment via letter using practice lists.

### Strengths and limitations

The main strength of this study was its factorial design to test two key uncertainties in the conduct of a future RCT, that is, the method of random allocation (cluster versus individual) and the method of participant recruitment (opportunistic versus systematic). However, we recognise that our study has some limitations.

One limitation of this feasibility study was the absence of any objective measure of internal validity that would have helped to inform whether individual or cluster randomisation was more appropriate, although the attrition may provide insight into this issue, with greater attrition threatening internal validity. Given the additional sample size required in cluster randomised trials, such a measure would have been helpful. In addition, although we randomised participants and practices to design factors in order to minimise imbalance in the number of participants across the various design factors, their permutations were not completely balanced. Our statistical efficiency to detect differences in the feasibility outcomes across the four design factor groupings was probably reduced by unequal participant numbers across the groupings. Furthermore, no specific measures of the feasibility or fidelity of implementing the intervention were employed.

The use of the pedometer as the means of collecting the primary outcome as well as being part of the intervention (Arm C) may be problematic, as the fidelity of the intervention was linked with the fidelity of the data captured. Furthermore, we were unable to ensure that participants who received a sealed pedometer (Arms A and B) did not in fact unseal the pedometer to check their readings prior to the scheduled day for data collection. An alternative method of capturing physical activity, such as an accelerometer, may have been beneficial, and would have captured other forms of physical activity, and more detailed data about the activity, such as intensity. This may be particularly relevant within this study as the control arm received no advice about walking specifically, and therefore may have engaged in other forms of activity not recorded fully by the pedometer. Due to the lack of full power regarding the primary outcome, we were unable to make any specific comments comparing blinded and unblinded pedometer use on physical activity.

The experiences of GPs in delivering the interventions, fidelity of the intervention delivery, and issues surrounding contamination with individual randomisation were qualitatively assessed and are not reported here.

## Conclusions

Overall, researchers in primary care should consider how practice characteristics and methods of recruiting participants can influence the characteristics of the sample, as well as the potential differences in time to complete recruitment of the required participant sample size. Our results indicate that participants recruited opportunistically may be more likely to withdraw, implying that increased numbers are required to attain a specific sample size when taking attrition into consideration; the requirement to recruit more participants initially may be offset by more rapid recruitment compared with systematic means (for example by letter). However, demographic characteristics of participants may differ by recruitment method, possibly implying that a mixture of both methods may best facilitate a wide demographic base and a reasonable recruitment rate. Our study was small and required the active involvement of participants who were not being treated within the trial for a specified medical condition; rather, the trial interventions aimed to change the physical activity behaviour of participants whose health states may have varied widely, from those who were fundamentally well to those who may have been in poorer health. Hence, our findings may not be generalisable to trials aimed at treating a specified medical condition, and there is a need for further pilot trials in the primary care setting to compare different methods of recruitment and randomisation.

## Abbreviations

CI: confidence interval; DDELPHI: Doctor-DELivered PHysical activity Intervention; EOI: expression of interest; EQ-5D: European Quality of Life (EuroQoL)-5 dimensions; GP: general practitioner; GPPAQ: General Practice Physical Activity Questionnaire; ICC: intra-cluster correlation coefficient; IMD: Index of Multiple Deprivation; NHS: National Health Service, NICE, National Institute for Health and Care Excellence (formerly National Institute for Health and Clinical Excellence); NIHR: National Institute for Health Research; PCRN: primary care research network; RCT: randomised controlled trial; UK: United Kingdom.

## Competing interests

The authors declare that they have no competing interests.

## Authors’ contributions

MH designed and oversaw the study. FCW performed the statistical analyses in collaboration with RST and prepared the first draft of the manuscript. KS, SB, and CH recruited the practices and participants, and collected the data. RST, JLC, MT, MM, DS, KMT, AS and TAH contributed to the overall design and conception of the study. All authors commented on earlier drafts and approved the final version of the manuscript. All authors were members of the study steering committee that oversaw the management of the study.
